# Protection against Pertussis in Humans Correlates to Elevated Serum Antibodies and Memory B Cells

**DOI:** 10.3389/fimmu.2017.01158

**Published:** 2017-09-15

**Authors:** Valentina Marcellini, Eva Piano Mortari, Giorgio Fedele, Francesco Gesualdo, Elisabetta Pandolfi, Fabio Midulla, Pasqualina Leone, Paola Stefanelli, Alberto Eugenio Tozzi, Rita Carsetti, The Pertussis Study Group, E. Agricola

**Affiliations:** ^1^B Cell Physiopathology Unit, Immunology Research Area, Bambino Gesù Children’s Hospital, IRCSS, Rome, Italy; ^2^Department of Infectious, Parasitic and Immune-Mediated Diseases, National Institute of Health, Rome, Italy; ^3^Multifactorial Disease and Complex Phenotype Research Area, Bambino Gesù Children’s Hospital, IRCSS, Rome, Italy; ^4^Department of Pediatrics, University of Rome “La Sapienza”, Rome, Italy; ^5^Diagnostic Immunology Unit, Department of Oncohematology, Bambino Gesù Children’s Hospital, IRCSS, Rome, Italy

**Keywords:** pertussis, vaccination, immune system, sIgA, memory B cells

## Abstract

Pertussis is a respiratory infection caused by *Bordetella pertussis* that may be particularly severe and even lethal in the first months of life when infants are still too young to be vaccinated. Adults and adolescents experience mild symptoms and are the source of infection for neonates. Adoptive maternal immunity does not prevent pertussis in the neonate. We compared the specific immune response of mothers of neonates diagnosed with pertussis and mothers of control children. We show that women have pre-existing pertussis-specific antibodies and memory B cells and react against the infection with a recall response increasing the levels specific serum IgG, milk IgA, and the frequency of memory B cells of all isotypes. Thus, the maternal immune system is activated in response to pertussis and effectively prevents the disease indicating that the low levels of pre-formed serum antibodies are insufficient for protection. For this reason, memory B cells play a major role in the adult defense. The results of this study suggest that new strategies for vaccine design should aim at increasing long-lived plasma cells and their antibodies.

## Introduction

Mammalians protect their progeny during the first days and months of life through the adoptive transfer of maternal antibodies. At birth, the still immature immune system of the newborn faces the challenge of encountering thousands of different commensal and pathogenic microorganisms, which rapidly colonize its body surfaces and mucosal epithelia. In this phase, the adoptive transfer of the maternal immune experience helps the newborn to survive and generate its own immune defenses (1). Before birth, the mother transfers to the fetus her systemic memory, represented by serum IgG, which will protect the child during the first months of life, although rapidly decaying between three and six months of age (2). After birth, the neonate can only receive the mucosal immune memory of the mother through the IgA antibodies transferred with the breast milk. Human milk not only contains all the nutrients and vitamins needed for neonatal growth and development, but it is also rich of substances with anti-infective activity, which protect the neonate from the microorganisms colonizing the respiratory ways and gut immediately after birth (3, 4). Whereas cytokines, defensins, and lactoferrin, produced by the innate immune system of the mother, exert a wide-range protection, secretory IgA defend the child only from pathogens known by the immune system of the mother (5). Milk IgA derives from plasma cells that migrate from mucosal sites to the mammary gland at the end of pregnancy and during lactation. Migration is driven by chemokines and mediated by adhesion molecules (6, 7). Gut IgA is generated by T-cell-dependent (TD) and -independent mechanisms (8). Most commensal bacterial species are coated by polyreactive T-cell-independent natural IgA. Natural IgA has the additional function of favoring bacterial uptake by Peyer patches where the TD adaptive immune response generates highly specific IgA. Similar to natural IgA at mucosal sites, natural IgM in the serum has not only the function of first-line defense against infection but also the function of trapping antigen into complexes that are rapidly delivered to follicular dendritic cells to initiate and maintain the germinal center (GC) reaction. Natural IgM in the serum is produced by IgM memory B cells. IgM memory B cells, also called “natural/natural effector” or innate memory B cells, can be found in patients unable to form GCs (9–11), suggesting that they have a T-cell-independent origin (12–14). The other type of memory B cells in the peripheral blood, switched memory B cells, is generated by TD adaptive immune responses in the GC (14). One-third of the switch memory B cells express IgA, and the rest are of IgG isotype. IgA and IgG switched memory B carry many somatic mutations (SM) and are highly specific. We have recently shown that innate memory B cells revise their B-cell receptor in the GC acquiring SM and becoming remodeled IgM memory B cells (14).

Vaccinations, similar to natural infections, trigger the GC reaction resulting in the generation of two cell types, both expressing high-affinity antibodies: long-lived memory plasma cells and switched memory B cells. Memory plasma cells continuously secrete their antibodies thus ensuring the presence of pre-formed specific antibodies in the serum. Memory B cells instead are the main actors of the recall response. They rapidly react to a renewed antigen encounter with proliferation and plasma cells formation to increase the concentration of specific antibodies and prevent re-infection and disease (15).

In order to study the *in vivo* response to infection, we analyzed peripheral blood, serum, and breast milk of women exposed to pertussis infection.

Pertussis is a respiratory infection caused by *Bordetella pertussis* that may be particularly severe and even lethal in the first months of life especially when infants are still too young to be vaccinated. Breast feeding protects the neonate from several respiratory and gastro-enteric pathogens, as demonstrated by the observation that the mortality rate due to infection is reduced by half in neonates receiving maternal milk (5, 16). Based on the results of a case–control study, however, we have recently demonstrated that breast feeding does not protect infants from pertussis (17). In addition, we have shown that mothers may represent the source of infection for children in 50% of the cases (18).

In order to understand why the maternal immune system is unable to prevent neonatal pertussis infection, we conducted a study on the mothers of infants (<6 months) diagnosed with pertussis and compared them to two groups of controls. The first group included mothers of healthy infants admitted as outpatients for hip ultrasound screening [healthy controls (HCs)] and the second mothers of children hospitalized for lower respiratory tract infections (LRTI) other than pertussis.

We found that mothers that are not infected or exposed to pertussis (HC and LRTI) have low levels of pertussis-specific antibodies in the serum and breast milk and are therefore unable to transfer passive protection to their children before or after birth. Mothers of children with pertussis actively protect themselves from the ongoing infection by increasing the number of specific memory B cells that secrete IgG and IgA and producing anti-pertussis toxin IgG and IgA antibodies in the serum.

IgM memory B cells secreting antibodies able to react with pertussis antigens and *Bordetella*-binding milk IgA increase, not only in mothers of children with pertussis but also in the mothers of neonates with LRTI. Thus, whereas switched memory B cells and serum IgA and IgG increase specifically in response to pertussis, IgM memory B cells and milk IgA are aspecifically induced during infection generating a wide spectrum antibody response.

## Patients and Methods

### Study Population

In this study, we included the mothers of infants enrolled in a case–control investigation conducted between June 2012 and January 2015 in two Italian large metropolitan pediatric hospitals, located in Rome, Italy. Part of the results of this study was published before (17). Mothers included in the study were mothers of unvaccinated children, younger than 6 months, hospitalized for pertussis, diagnosed by real-time polymerase chain reaction. Mothers of two groups of controls aged <6 months were enrolled including healthy infants admitted as outpatients for hip ultrasound screening at one of the participating hospitals or infants hospitalized because of low tract respiratory infections other than pertussis, in the same period during which cases were recruited. Mothers of enrolled children were requested to undergo a whole blood and serum sample collection and to donate a sample of breast milk.

Information on the medical histories of the eligible parents was collected onto standard report forms. Data were collected through a questionnaire administered to parents of patients (pertussis, LRTI and HC) at enrollment, after signing an informed consent.

Epidemiological, microbiological, and immunological data were recorded in a unique electronic database. The median age of mothers was 34.6 years (range 18.4–46.4). Among these, 15% were smokers, 33% were graduates, and 65% had a job. None had been vaccinated against pertussis and no one remembered having had the disease.

The study was approved by the Bambino Gesù Children’s Hospital Ethical Committee (protocol no RF-2010-2317709). Informed consent was signed from mothers of children and the study was performed following the guidelines of the Declaration of Helsinki.

### Samples

Sociodemografic, clinical, and microbiological data of children and families included in the study were published elsewhere (17). Only mothers who were breast feeding were included in the immunological study. We collected blood samples from 61 HC mothers (HC), 36 mothers of children with LRTI other than pertussis (LRTI) and 57 mothers of children with pertussis (PERTUSSIS). Milk was obtained from 61 HC, 21 LRTI, and 53 PERTUSSIS women. Serum anti-PT levels were available for 50 of the HC, 17 of the LRTI, and 57 of the PERTUSSIS mothers included in the immunological study.

None of the enrolled mothers had been vaccinated against pertussis or recalled to have had the disease.

### Analysis of Breast Milk

Whole-milk aliquots were stored at −20°C in a frost-free freezer, until assayed for pertussis-specific antibodies.

### Bacteria Preparation for Live Bacterial FACS

Bacterial isolates were cultured on Columbia agar + 5% sheep blood for 24 h at 37°C, morphologically characterized and subsequently identified with matrix-assisted laser desorption ionization time-of-flight mass spectrometry.

*Bordetella pertussis* isolated from nasopharyngeal aspirates was stored at −80°C, using a system of small glass spheres in a test tube from freezing Cryobank (Mast Diagnostics GmbH).

To obtain viable bacteria, a sphere with adherent bacteria was removed from the frozen test tube using a sterile needle and immediately placed on Bordetella Selective Agar (Biolife Italian Srl, Italy), where the bead was streaked on the surface of the soil to distribute the bacterial cells and incubated at 35°C in a humid atmosphere for approximately 4–5 days.

Bacterial colonies were collected using a sterile swab and mixed in a test tube with a sterile saline solution of NaCl 0.45% (Bio Merieux S.A.) to obtain a suspension of 3 McFarland.

### Live Bacterial FACS

The study included *B. pertussis* and other seven frozen barcoded bacterial isolates from routine specimens (*Klebsiella pneumoniae, Pseudomonas aeruginosa, Streptococcus pneumoniae, Streptococcus salivarius, Staphylococccus aureus, Enterococcus faecalis*, and *Escherichia coli*).

Bacterial single colonies and *B. pertussis* suspension were diluted in 1.5 μL PBS. For each staining, 45 μL of bacteria suspension was incubated with either FACS buffer (PBS + FBS 2% + sodium azide 0.1%) (negative control) or maternal milk (5 μL of 1:10, 1:100 dilution in FACS buffer) and left for 20 min on ice. Samples were washed three times with ice-cold FACS buffer and then anti-human IgA-fitc-labeled was added for 20 min. After three washing steps, bacteria were diluted in 300 μL ice-cold FACS buffer and data was acquired on a FACS Canto II (BD Bioscience) using FCS and SSC parameters in logarithmic mode (Figures S1A,B in Supplementary Material). Data were analyzed using the DIVA 6.0 software (BD Bioscience).

The binding of human IgA to bacteria (bacterial binding) was measured by comparing the staining for IgA of each bacterial species incubated either with FACS buffer or with milk. The frequency of bacterial binding is a semi-quantitative measure of the concentration of specific antibody in the analyzed sample.

### ELISA

Pertussis toxin-specific IgG (PT-IgG) was measured in sera of mothers using the ELISA standardized within a European Sero-Epidemiology Network (19, 20) as detailed elsewhere (21). In-house reference sera and international standards (06/142, WHO International Standard Pertussis Antiserum) were calibrated against the USA-FDA standard serum (lot 3-HRP3) (22). The results were expressed in international units (IU)/mL. The limit of detection of the assay was 1 IU/mL. Pertussis toxin-specific IgA (PT-IgA) was measured in sera using a commercial ELISA kit (Anti-Bordetella Pertussis Toxin IgA, Euroimmun AG) according to the manufacturer’s instruction. Data are expressed in IU/mL, and the lower detection limit was 0.7 IU/mL.

Pertussis toxin-specific IgM (PT-IgM) was measured in sera using an in-house ELISA. Briefly, Immulon^®^ Microtiter™ 96-Well Plates (Termofisher) coated with 200 ng of purified PT antigen (21) were incubated with 100 mL/well of 1:100 prediluted sera for 2 h at 28°C. After washing, peroxidase-conjugate anti-human IgM Ab (Jackson Immuno Research Laboratories) was added to the wells and plates incubated O.N. at RT. After incubation, the plates were washed and the substrate solution added (OPD; Sigma-Aldrich). The reaction was stopped after 20 min by the addition of 10% sodium dodecyl sulfate, and plates were read at 450 nm wavelength. Since no specific international standards for PT-IgM are available, optical density (OD) values are reported.

### Cell Isolation and Flow Cytometry B Cells Phenotype

Heparinized peripheral blood mononuclear cells (PBMCs) were isolated by Ficoll Paque™ Plus (Amersham Pharmacia Biotech) density-gradient centrifugation, counted, and stained with the appropriate combination of fluorescent labeled with anti-CD19, anti-CD27, anti-CD24, and anti-IgM antibodies and analyzed by flow cytometry. We calculated, in the lymphocyte gate, the frequency of total B (CD19^+^) and memory B cells (CD19^+^CD24^+^CD27^+^). We also measured the frequency of IgM (CD19^+^CD24^+^CD27^+^Ig^+^) and switched (CD19^+^CD24^+^CD27^+^IgM^−^) memory B cells in the memory B cells gate. For each staining, at least 50,000 events in the lymphocyte gate were collected on a FACS Canto II. Data were analyzed using the DIVA 6.0 software (BD Bioscience).

### CpG Stimulation and ELISPOT

Peripheral blood mononuclear cells were cultured in complete medium at a concentration of 2.5 × 10^6^ cells/mL. A total of 0.35 µM CpG B ODN2006 (Hycult Biotech) was added to stimulate the proliferation and differentiation of memory B cells for 5 days.

Ninety-six-well plates (MultiScreen-HA, Milipore) were coated overnight with AffiniPure F(ab′)2 Fragment Goat anti-human IgA^+^IgG^+^IgM (H + L; Jackson Immuno Research Laboratories) for the measurement of total memory B cells. For the detection of specific memory B cells, plates were coated with *B. pertussis* antigens (5 µg/mL): purified PT (NIBSC, Potters Bar, UK), filamentous hemagglutinin (FHA) (NIBSC, Potters Bar, UK), and pertactin (PRN) (List Biological Labs, Campbell, CA). After washing with sterile PBS/0.05% Tween 20, plates were blocked for 1 h at 37°C with PBS/gelatin 1%.

Peripheral blood mononuclear cells stimulated for 5 days, as described before, were collected, counted, and seeded in the pre-coated plates. Plates were left at 37°C, 2% CO_2_ for 4–6 h to allow antibody secretion. A total of three 1:2 serial dilutions were done starting in the first well with 5 × 10^4^ cells for detection of total IgM, IgG, and IgA. A total of 2 × 10^5^ cells were seeded in the first dilution well (three 1:2 serial dilutions) for the detection of B cells secreting specific antibodies. After incubation, plates were washed with dH_2_O/0.05% Tween 20 (once) and PBS/0.05% Tween 20 (two times) and incubated overnight with either anti-IgM HRPO (1:1,000), anti-IgG HRPO (1:2,000), or anti-IgA (1:2,000; Jackson Immuno Research Laboratories) diluted in PBS + gelatin (1 + 0.05%) Tween 20 (Sigma). After washing twice as before, TMB substrate (ready to use from Mabtech) was used according to the manufacturer’s instructions. Plates were left at room temperature to allow the blue color to develop and the reaction was stopped with dH_2_O. Plates were left to dry before counting with an ELISCAN (A-EL-VIS).

### Statistical Analysis

Data are presented as median (interquartile range). Mann–Whitney *U* test was used to compare all data because of distribution. Pearson correlation coefficient was used to measure the linear correlation between samples. GraphPad Prism software (CA, USA) was used for statistical analysis. A *p*-value of less than 0.05 was considered as statistically significant.

## Results

### Serum Antibodies

PT is a highly antigenic and specific component of *B. pertussis* (23). Anti-PT-IgG ≥ 100 IU/mL is used as specific indicator of recent pertussis infection (18).

**Figure 1:**
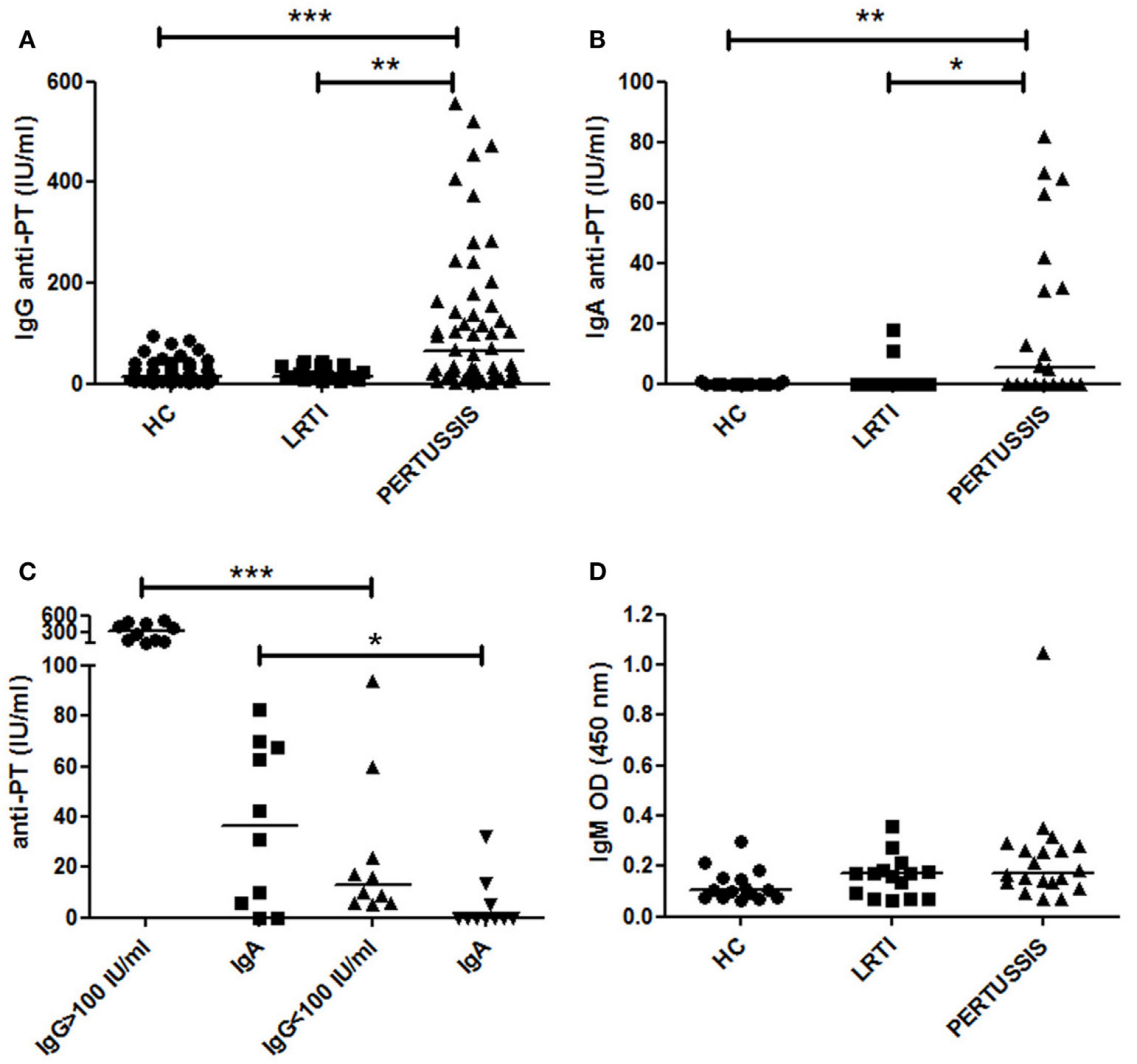
Anti-PT-specific Ig in the serum. **(A,B)** The plot shows the concentration of specific anti- pertussis toxin-specific IgG (PT-IgG) or pertussis toxin-specific IgA (PT-IgA) in the serum of control [healthy controls (HC) and lower respiratory tract infections (LRTI)] and PERTUSSIS mothers. **(C)** The plot shows the concentration of specific anti-PT-IgA in the serum of PERTUSSIS mothers with anti-PT-IgG ≥ 100 IU/mL or with anti-PT-IgG < 100 IU/mL. **(D)** Optical density value of IgM in the serum of control (HC and LRTI) and PERTUSSIS mothers. Statistical significance was determined using the Mann–Whitney test; *p < 0.05, **p < 0.01, and ***p < 0.001. A total of 50 HC, 17 LRTI, and 57 PERTUSSIS samples could be evaluated for IgG. A total of 15 HC, 15 LRTI, and 20 PERTUSSIS samples could be evaluated for IgA and IgM.

We measured IgG antibodies directed against PT in the serum of mothers of HC, LRTI, and PERTUSSIS infants by ELISA. IgG antibodies were present at significantly lower concentration in HC [13.45 (8.32–38.15) *p* ≤ 0.0001] and LRTI [14 (11–36) *p* = 0.002] mothers than in PERTUSSIS mothers [63.5 (16.75–158.3)] (Figure 1A).

Anti-PT-IgA concentration increases during *B. pertussis* infection but decays more rapidly compared to IgG. Cutoff value of 20 IU/mL is indicative of a recent pertussis infection (Prospective Evaluation of an Australian Pertussis Toxin IgG and IgA Enzyme Immunoassay). IgA against PT significantly increased in PERTUSSIS [5.5 (0–39.5)] mothers compared to HC [0 (0–0) *p* = 0.005] and LRTI [0 (0–0) *p* = 0.01] mothers (Figure 1B). There was a positive correlation between the concentration of IgG and IgA in the PERTUSSIS mothers (Pearson *r* = 0.4, *p* = 0.03).

In Figure 1C, we show that in the PERTUSSIS group, mothers with anti-PT-IgG ≥ 100 IU/mL had significantly higher level of anti-PT-IgA than mothers with anti-PT-IgG < 100 IU/mL, indicating an ongoing specific immune response against *B. pertussis* infection.

Anti-PT-IgM is not usually included in the tests for the diagnosis of pertussis, and no international standard is available. We performed an in-house ELISA using coated plates with purified PT antigen. We used OD values as a measure of IgM concentration. We found that anti-PT-IgM was increased not only in the serum of PERTUSSIS (*p* = 0.02) but also in the serum of LRTI mothers when we compared them to HC mothers (Figure 1D) indicating an aspecific increase in IgM during infection. Only one mother of the PERTUSSIS group had high IgM OD value, probably reflecting a primary immune response (IgG levels were 94 IU/mL in this individual).

In our study, we were not able to measure the variation of anti-PT-IgG titers before and after pertussis exposure in the same subject. We could only compare individual values of mothers of HC neonates and of children diagnosed with PERTUSSIS (Figure 1).

No correlate of protection has been established for pertussis, but anti-PT-IgG levels >5 IU/mL has been considered potentially protective (24). Eighty-four percent of HC mothers had antibody levels >5 IU/mL. As none of the mothers had ever been vaccinated against pertussis, or remembered to have had pertussis in the past, serum anti-PT-IgG can be considered as an indication of a previous undiagnosed infection. A significant increase in anti-PT-IgG was observed in the PERTUSSIS mothers demonstrating that the immune system is activated even in the presence of pre-formed antibodies (Figure 1).

The increase in IgG in the serum of mothers of PERTUSSIS group could be explained by the function of pre-existing memory B cells rapidly producing antibodies in response to bacterial challenge, thus performing a classical recall response.

### B-Cell Phenotype and Total Memory B Cells

**Figure 2:**
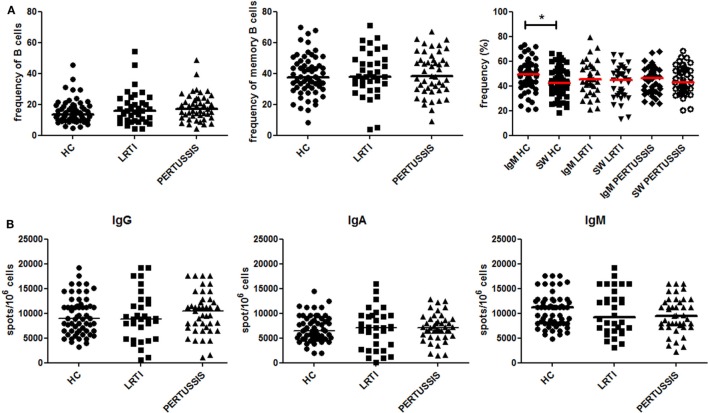
Peripheral B-cell subset analysis. **(A)** Frequency (%) of B cells (defined as CD19^+^), total memory B cells (CD19^+^CD27^+^), IgM (CD19^+^CD27^+^IgM^+^), and switched (CD19^+^CD27^+^IgM^−^) memory B cells is depicted for healthy controls (HC), lower respiratory tract infections (LRTI), and PERTUSSIS mothers. The staining was performed using antibodies anti-CD19, -CD24, -CD27, and -IgM. **(B)** A number of total IgM, IgA, and IgG spots per million of total peripheral blood mononuclear cells are shown both for control (HC and lower respiratory tract infections) and PERTUSSIS mothers. A total of 61 HC, 36 LRTI and 47 PERTUSSIS mothers were compared. Mann–Whitney statistical test was used for calculation of the reported *p*-value. Statistical significance is marked as *p < 0.05.

We compared the peripheral blood B-cell compartment of HC, LRTI, and PERTUSSIS mothers. There was no difference in the frequency of total B cells and memory B cells between the three groups (Figure 2A). In the memory pool, whereas PERTUSSIS and LRTI mothers had equal frequencies of IgM and switched memory B cells, IgM memory B cells were significantly more than switched memory B cells (*p* = 0.014) in HC mothers (Figure 2A).

The number of memory B cells in the peripheral blood can be determined also based on their ability to secrete antibodies, by ELISPOT. We stimulated PBMCs from mothers of control (HC and LRTI) and pertussis children with the TLR9 ligand CpG in order to induce the proliferation of memory B cells and their differentiation into antibody-producing cells (12, 25). We found comparable frequencies of memory B cells secreting IgM^−^IgA^−^ or IgG in HC, LRTI, and PERTUSSIS mothers (Figure 2B).

### Memory B Cells Secreting Specific Anti-Pertussis IgM, IgA, and IgG

We measured the frequency of memory B cells in the peripheral blood by ELISPOT against three different pertussis antigens (PT, FHA, and PRN). The number of antigen-specific spots was divided by the total number of total IgG spots in order to calculate the frequency of specific spots in the total pool of memory B cells.

**Figure 3:**
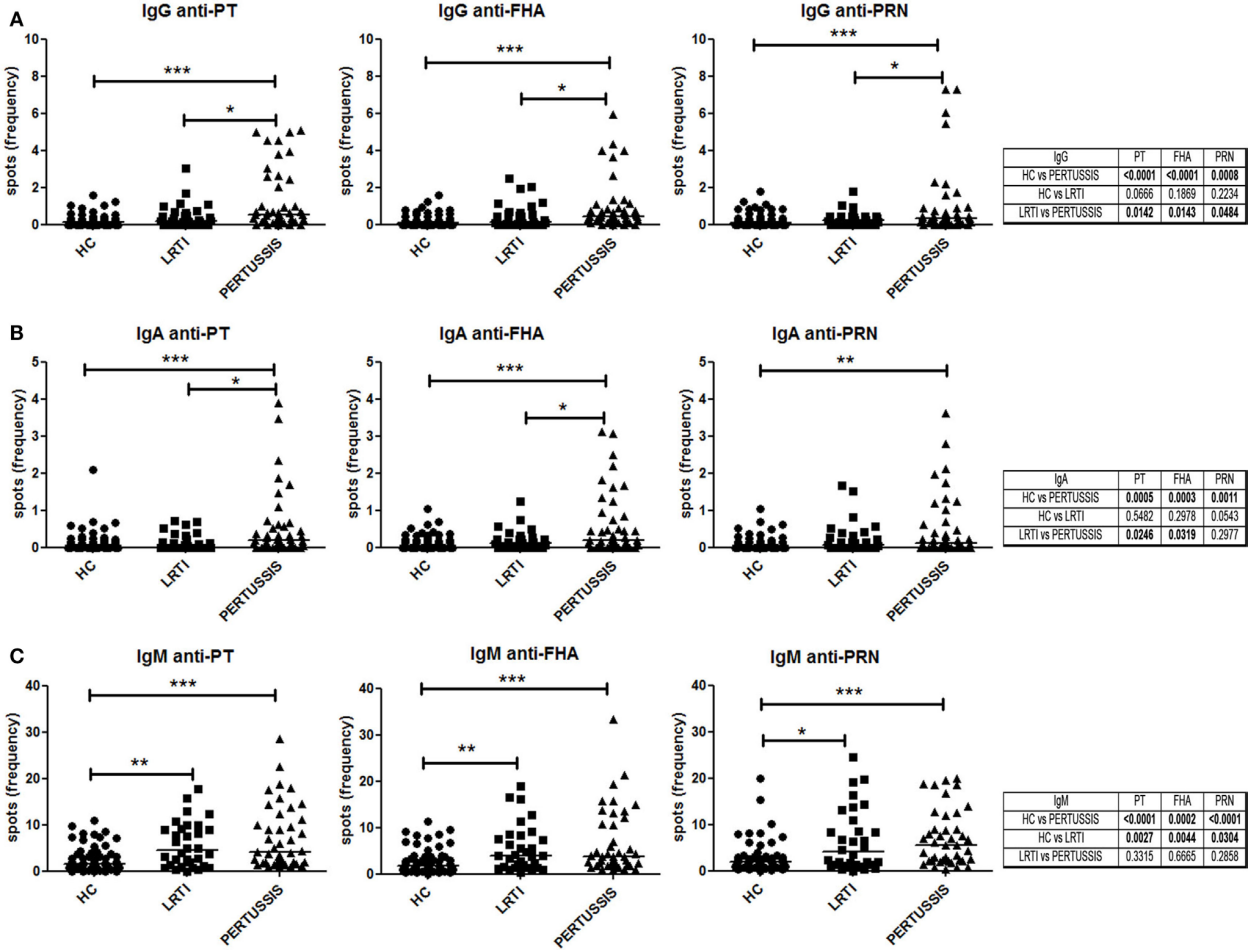
Memory B cells specific for pertussis antigens. **(A,B,C)** Number of specific anti-PT (toxin [PT], filamentous hemagglutinin [FHA], and pertactin [PRN])-IgG, -IgA, and -IgM spots per million of total cultured peripheral blood mononuclear cells are shown in healthy controls (*n* = 61), lower respiratory tract infections (*n* = 36), and PERTUSSIS (*n* = 47) mothers. Statistical significance was determined using the Mann–Whitney test; *p < 0.05, **p < 0.01, and ***p < 0.001.

Pertussis-specific switched memory B cells changed in response to infection. In the PERTUSSIS mothers, the number of B cells producing IgG against the three different antigens significantly increased compared to HC and LRTI mothers. No increase in memory B cells producing IgG against *B. pertussis* was observed in the LRTI group (Figure 3A, plots and Mann–Whitney *U* statistics).

We also measured serum IgA, because IgA is the major immunoglobulin produced in the respiratory tract at the site of *B. pertussis* invasion (26). We observed a significant increase in the frequency of the IgA spots against the three pertussis antigen between HC and PERTUSSIS mother (PT *p* = 0.0005; FHA *p* = 0.0003; PRN *p* = 0.001). Memory B cells secreting IgA against PRN were slightly increased in the mothers of children with LRTI (Figure 3B).

Thus, mothers of neonates hospitalized because of pertussis were actively protecting themselves against the pathogen by increasing the frequency of specific memory B cells.

As previously observed for other bacterial antigens, the frequency of IgM spots is higher than those of IgA and IgG isotype (27). B cells producing IgM against pertussis increased significantly between HC and PERTUSSIS mothers (PT and PRN *p* ≤ 0.0001; FHA *p* = 0.0002), but, in contrast to switched memory B cells, IgM memory B cells able to bind pertussis antigens also increased significantly in mothers of children with LRTI (PT *p* = 0.002; FHA *p* = 0.004; PRN *p* = 0.03) (Figure 3C). Thus, during respiratory infections (LRTI and PERTUSSIS), IgM increases non-specifically.

### IgA in the Milk

**Figure 4:**
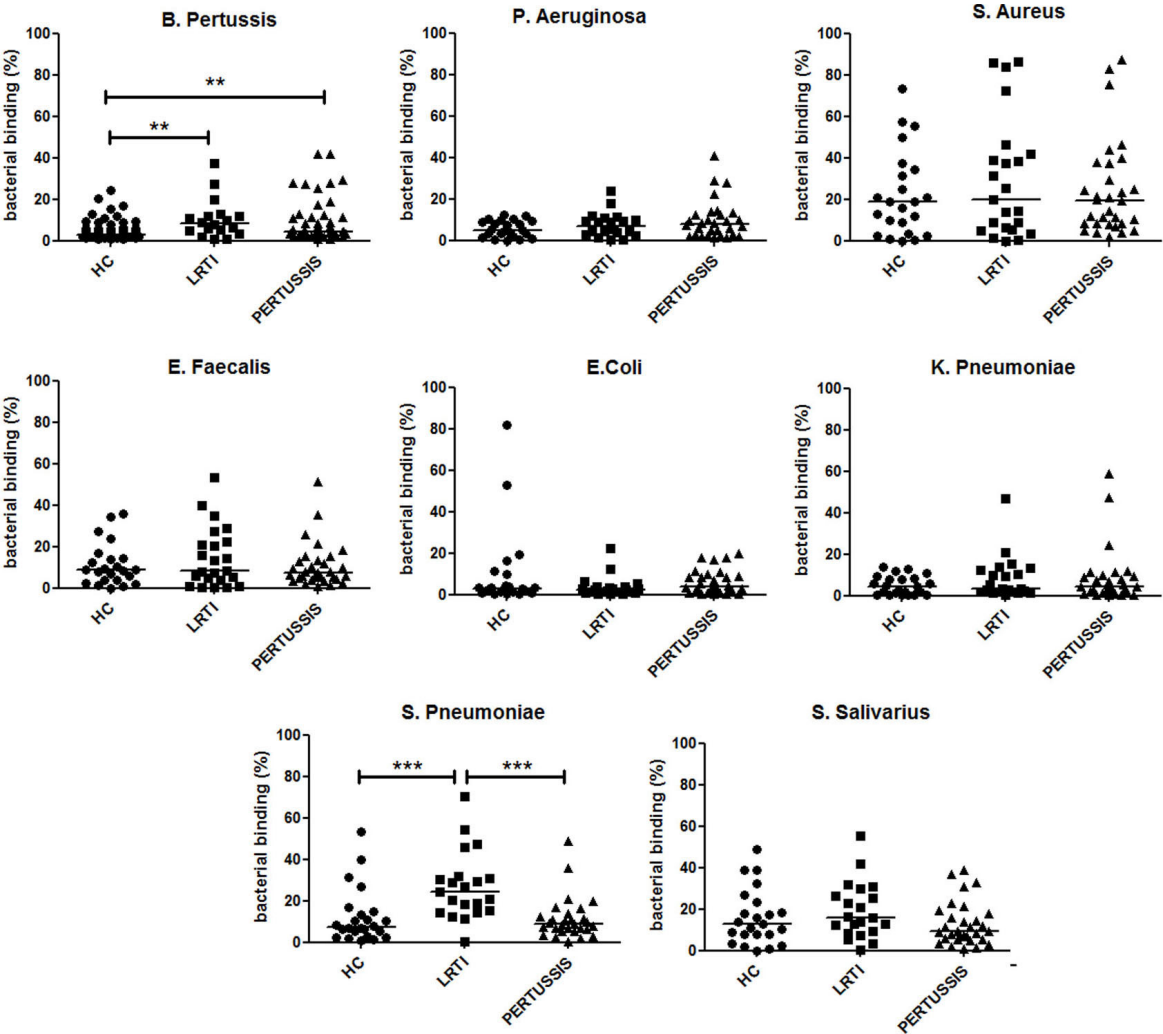
IgA in breast milk reacts with several bacterial species. Graphs show the frequency of bacteria of the indicated species binding to IgA contained in the milk (1:10 dilution) of healthy controls (HC), lower respiratory tract infections (LRTI), and PERTUSSIS mothers. The difference between groups was determined using the Mann–Whitney test; **p < 0.01 and ***p < 0.001. Milk from 61 HC, 21 LRTI, and 53 PERTUSSIS mothers was analyzed for binding to *B. pertussis*, and 24 HC, 21 LTR and 28 PERTUSSIS samples were tested against all other bacteria.

After birth, the neonate can only receive the mucosal immune memory of the mother through the IgA antibodies transferred with breast milk. We detected the abundance of IgA reacting with eight different bacteria (*B. pertussis, S. pneumoniae, K. pneumoniae, Escherichia coli, Enterococcus faecalis, Streptococcus salivarius, Staphylococcus aureus*, and *Pseudomonas aeruginosa*) in breast milk of HC, LRTI, and PERTUSSIS. We measured the ability of milk IgA to react with fresh bacteria using the bacterial FACS technique (Figures S1A,B in Supplementary Material). We observed that breast milk of HC, LRTI and PERTUSSIS mothers contains IgA able to react with different commensal and pathogenic microorganisms that can be found in the microbial communities colonizing airways, gut, and skin. IgA reacting with *B. pertussis* was present in low amounts compared to IgA against other bacterial species (Figure S1C in Supplementary Material). In the milk of PERTUSSIS mothers IgA antibodies reacting with *B. pertussis* were significantly increased compared to HC mothers (*p* = 0.001) (Figure 4). IgA anti-pertussis was also increased in LRTI mothers (*p* = 0.003). In this group, also IgA directed to *S. pneumoniae* was significantly higher than in HC or PERTUSSIS mothers (Figure 4). No significant difference was found in the amount of antibodies binding other bacterial species between the milk of the three groups (Figure 4).

## Discussion

Pertussis is a severe and even lethal disease for children too young to be vaccinated. It has been shown that in the majority of the cases neonates are infected by their mothers or by household members. Awareness among physicians about pertussis circulation has led to increased suspect of the disease in patients with persistent coughing and, consequently, to a more frequent testing. It has been shown that *B. pertussis* infects subjects of all ages who act as infection source causing local clusters of disease (28). Adults and adolescents may be unaware of being infected by *B. pertussis* as they experience mild symptoms, difficult to distinguish from those caused by other respiratory infections. Mothers included in our study had never been vaccinated and did not recall a previous pertussis infection. Based on the high concentration of anti-PT-IgG in the serum, we have hypothesized that they were the source of infection for their children in at least 50% of the cases (18). We now show that, although symptoms were modest or absent, the immune system of the PERTUSSIS mothers actively reacted against the pathogen by expanding antigen-specific memory B cells and thereby increasing the production of antibodies of IgG and IgA isotype. Thus, we hypothesize that, in the adult, specific memory B cells prevent severe clinical pertussis by rapidly producing antibodies. The detectable baseline levels of antibodies and memory B cells in control mothers (HC and LRTI) and the ability to generate a recall response, demonstrated in PERTUSSIS mothers, indicate that previous contacts with *B. pertussis* have occurred in the adult population. These subclinical contacts generated an immune memory that is sufficient to prevent a severe disease but not to avoid colonization, disease, and transmission.

Serum antibodies are excellent correlates of protection for many infectious diseases (29) but for pertussis there is no “universally accepted quantifiable serological measure of protection” (30). We show that the recall response of memory B cells is used by the adult immune system for protection.

Maternal vaccination prevents neonatal pertussis. Retrospective studies showed 91% effectiveness of DTaP vaccination of mothers for protecting newborns against pertussis in the first 2 months of life (31, 32). Cocoon strategies involving the vaccination of both parents immediately after the delivery are instead unable to prevent pertussis in the neonate (33, 34). As the mother can exclusively transfer antibodies and no memory B cells during pregnancy, the effectiveness of maternal vaccination indicates that passively transferred IgG indeed protect against infection, but only at high concentrations. Placental transfer of anti-PT maternal antibodies is a highly efficient mechanism ensuring that specific IgG levels are higher in the newborn than in the mother (23). Transferred antibodies rapidly decay and at 2 months of age the concentration of anti-PT-IgG is decreased by 76% from the levels measured in cord blood (24).

After birth, the immune system of the mother can still contribute to the protection of the neonate through IgA antibodies in the milk. In breast milk, we find IgA antibodies binding bacterial species that are members of the microbial communities of the respiratory and intestinal mucosa and of the skin. IgA able to react with pertussis is present at low concentrations in breast milk of women not exposed to the infection and increased in mothers of the PERTUSSIS group. Thus, the low levels of *B. pertussis*-specific antibodies, in the serum and in the milk, explain why neonates are born without sufficient protection and cannot be helped to fight infection by breast feeding. Milk IgA derives from plasma cells that migrate from mucosal sites to the mammary gland at the end of pregnancy and during lactation. IgA able to bind *B. pertussis* is increased in the milk of mothers not only of the pertussis group but also in LRTI mothers indicating that infections induce a wide spectrum reaction at mucosal sites. Here, both natural and specific IgA are found. Natural IgA, produced by a T-cell-independent mechanism in the absence of intentional immunization, plays a similar function of natural IgM in the serum (35, 36).

Natural IgM is produced by IgM memory B cells. Here, we show that IgM memory B cells secreting antibodies against PT, FHA, and PRN are significantly increased in the peripheral blood not only in the mothers of the pertussis children but also in the LRTI group. The aspecific increase in IgM memory B cells probably reflects the nature of this population.

We have proposed that IgM and switched memory B cells have separate functions during infection (14, 15). IgM memory B cells play the role of first-line protection, whereas switched memory B cells, generated by the specific immune response in the GCs, eliminate the pathogen and remain in the organism to prevent re-infection. The IgM memory response observed not only in the mothers of the PERTUSSIS group but also in those of the LRTI group confirms that IgM memory B cells expand polyclonally as a first-line defense to infection. Accordingly, anti-PT-IgM increased in the serum of both PERTUSSIS and LRTI mothers. The increase in IgA in the milk of mothers of the PERTUSSIS and LRTI groups raises the question of whether polyclonal first-line reactions also occur at mucosal sites (35, 36).

Two important conclusions derive from our study, one concerning the protection of the neonate and the second concerning the relative roles of pre-formed antibodies and memory B cells.

We show that unvaccinated mothers cannot protect their children from pertussis because of the low levels of specific antibodies in the serum and breast milk. It is now clear that the best strategy to prevent the disease in infants is maternal vaccination during pregnancy. Acellular vaccines induce high levels of specific IgG in the serum of the mother, which will cross the placenta and prevent infection of the child (31, 32). Maternal vaccination effectively protects the neonate especially in the first two months after birth (32). The protective effects of maternal vaccination are not observed any more at 6 months of age (37). Passively transferred antibodies are highest in cord blood but rapidly decline and are much lower at two months of age (38), thus explaining the loss of the protective effect of maternal immunization on the infant.

Vaccine administration in pregnancy is safe for both mother and fetus. There is an interference of maternal IgG on the infant response to vaccination. A slight reduction of specific antibody titers in the children of immunized mothers has been observed after the first vaccine dose. The modest interference did not persist and disappeared after the following doses (39). Thus, maternal immunization does not impair the effectiveness of childhood vaccination. The optimal timing of vaccine administration during pregnancy has not yet been established. Third trimester vaccination results in high antibodies transfer to the neonate (39, 40), but second trimester vaccination is most effective for preterm neonates (41).

In our case–control study, based on serological data, we hypothesize that up to 50% of infants had probably been infected by the mother (18). If these mothers had been vaccinated in pregnancy, probably they would have not transmitted the disease and their children, having received high levels of maternal antibodies, would not have been hospitalized because of pertussis.

Several studies have started to address the issue of gender differences in the immune response and in immune-mediated diseases, demonstrating that the immune system of women is more active than that of men, responding better to infection, with the side effect of a higher incidence of autoimmune diseases (42, 43). The ability of women to produce more antibodies is useful for the survival of the species, because, in order to protect the neonate from infection in its first days and months of life, mothers donate to the child their antibodies of IgG and IgA type. This system is imperfect for pertussis and should be improved by maternal vaccination (44).

As PT is an important virulence factor for *B. pertussis* that damages the respiratory epithelium and impairs innate immune responses in the first few hours after infection, the low levels of pre-formed neutralizing antibodies explain why pertussis circulates notwithstanding the successfully implemented global vaccination strategies. On the other side, the mildness of the adult infection demonstrates that memory B cells are able to limit the damage and eliminate the pathogen. Memory B cells, generated by natural infection and vaccination, are indispensable for the rapid production of IgG antibodies during recall responses. There is no direct correlation between the number of memory B cells and the concentration of serum antibodies. In the case of hepatitis B vaccination, we have shown that memory B cells persist when antibodies decline below the protective threshold (45) and effectively respond to booster vaccine doses. Serum antibodies increase 3–7 days after triggering the recall response of memory B cells. For this reason when pre-formed serum antibodies are low, the ability of memory B cells to protect from an infectious disease depends on the nature of the pathogen (46). Hepatitis B has a slow pathogenesis, and memory B cells have enough time to differentiate into antibody secreting cells to ensure protection. In contrast, the pathogenesis of diseases caused by *Neisseria meningitides, S. pneumoniae*, and *Haemophilus influenzae* is so rapid (hours) that only pre-formed antibodies are able to protect the organism (46). Our results show that pertussis infection may be considered of intermediate pathogenicity, because memory B cells avoid a severe disease in the adult, but are not able to prevent all symptoms and most importantly do not block disease transmission.

It has been suggested that pertussis vaccines may be improved by better preserving the native structure of the antigens used for immunization (47) and including adjuvants designed to potentiate T-cell responses (48).

Our findings contribute to the discussion demonstrating that serum antibodies but not memory B cells are rapidly lost after natural infection. For this reason, more effective vaccines should also include adjuvants able to increase the number of specific long-lived plasma cells.

## Ethics Statement

The study protocols and consent forms were approved by the Ethical Committee of Ospedale Pediatrico Bambino Gesù, Rome, Italy. Informed consent was obtained from mothers of children and the study was performed following the guidelines of the Declaration of Helsinki.

## The Pertussis Study Group

The Pertussis Study Group members are as follows: **E. Agricola**, Multifactorial Disease and Complex Phenotype Research Area, Bambino Gesù Children’s Hospital, IRCSS, Rome, Italy; **C. M. Ausiello**, Department of Infectious, Parasitic and Immune-Mediated Diseases, National Institute of Health, Rome, Italy; **G. Buttinelli**, Department of Infectious, Parasitic and Immune-Mediated Diseases, National Institute of Health, Rome, Italy; **I. Campagna**, Multifactorial Disease and Complex Phenotype Research Area, Bambino Gesù Children’s Hospital, IRCSS, Rome, Italy; **C. Concato**, Virology Unit, Bambino Gesù Children’s Hospital, IRCSS, Rome, Italy; **F. Del Chierico**, Human Microbiome Unit, Bambino Gesù Children’s Hospital, IRCSS, Rome, Italy; **G. Di Mattia**, Department of Pediatrics, University of Rome “La Sapienza”, Rome, Italy; **B. Ferretti**, Multifactorial Disease and Complex Phenotype Research Area, Bambino Gesù Children’s Hospital, IRCSS, Rome, Italy; **A. Frassanito**, Department of Pediatrics, University of Rome “La Sapienza”, Rome, Italy; **M. V. Gonfiantini**, Multifactorial Disease and Complex Phenotype Research Area, Bambino Gesù Children’s Hospital, IRCSS, Rome, Italy; **R. Nenna**, Department of Pediatrics, University of Rome “La Sapienza”, Rome, Italy; **A. Nicolai**, Department of Pediatrics, University of Rome “La Sapienza”, Rome, Italy; **M. Onori**, Virology Unit, Bambino Gesù Children’s Hospital, IRCSS, Rome, Italy; **L. Putignani**, Human Microbiome Unit, Bambino Gesù Children’s Hospital, IRCSS, Rome, Italy; Parasitology Unit, Bambino Gesù Children’s Hospital, IRCSS, Rome, Italy; **C. Rizzo**, National Center for Epidemiology Surveillance and Health Promotion, National Institute of Health, Rome, Italy; **L. Russo**, Multifactorial Disease and Complex Phenotype Research Area, Bambino Gesù Children’s Hospital, IRCSS, Rome, Italy; **V. V. Spuri**, Virology Unit, Bambino Gesù Children’s Hospital, IRCSS, Rome, Italy; **L. Tanturri**, Department of Radiology, Bambino Gesù Children Hospital IRCSS, Rome, Italy; **A. Villani**, Department of Pediatrics and Infectious Disease, Bambino Gesù Children’s Hospital, IRCSS, Rome, Italy.

## Author Contributions

VM and PL performed the experiments. RC, EPM, GF, EP, FM, PS, FG, and AT designed the experiments and wrote the paper. The Pertussis Study Group collected the samples and the bacteria.

## Conflict of Interest Statement

The authors declare that the research was conducted in the absence of any commercial or financial relationships that could be construed as a potential conflict of interest.

## Supplementary Material

Figure S1 Description of the bacterial FACS method. **(A)** Colonies of bacterial isolates were diluted in FACS buffer and incubated either with FACS buffer or with maternal milk (1:10 and 1:30 dilutions). After washing, IgA bound to bacterial cells was revealed by a secondary staining with FITC-labeled mouse anti-human IgA. **(B)** Representative histograms showing the fluorescence of bacteria, without or with bound maternal IgA. **(C)** Relative representation of IgA binding to the indicated bacteria in the milk of healthy controls (HC), lower respiratory tract infections (LRTI), and PERTUSSIS mothers. As compared to *B. pertussis*, significantly higher amounts of antibodies bound to *S. Aureus, S. salivariu*s, and *S. pneumoniae* in all of the three groups.
