# Supplementary figures and images for: Protection against Pertussis in Humans Correlates to Elevated Serum Antibodies and Memory B Cells
Source: Front Immunol. 2017 Sep 15;8:1158. doi: 10.3389/fimmu.2017.01158 (PMC5605623; doi:10.3389/fimmu.2017.01158)

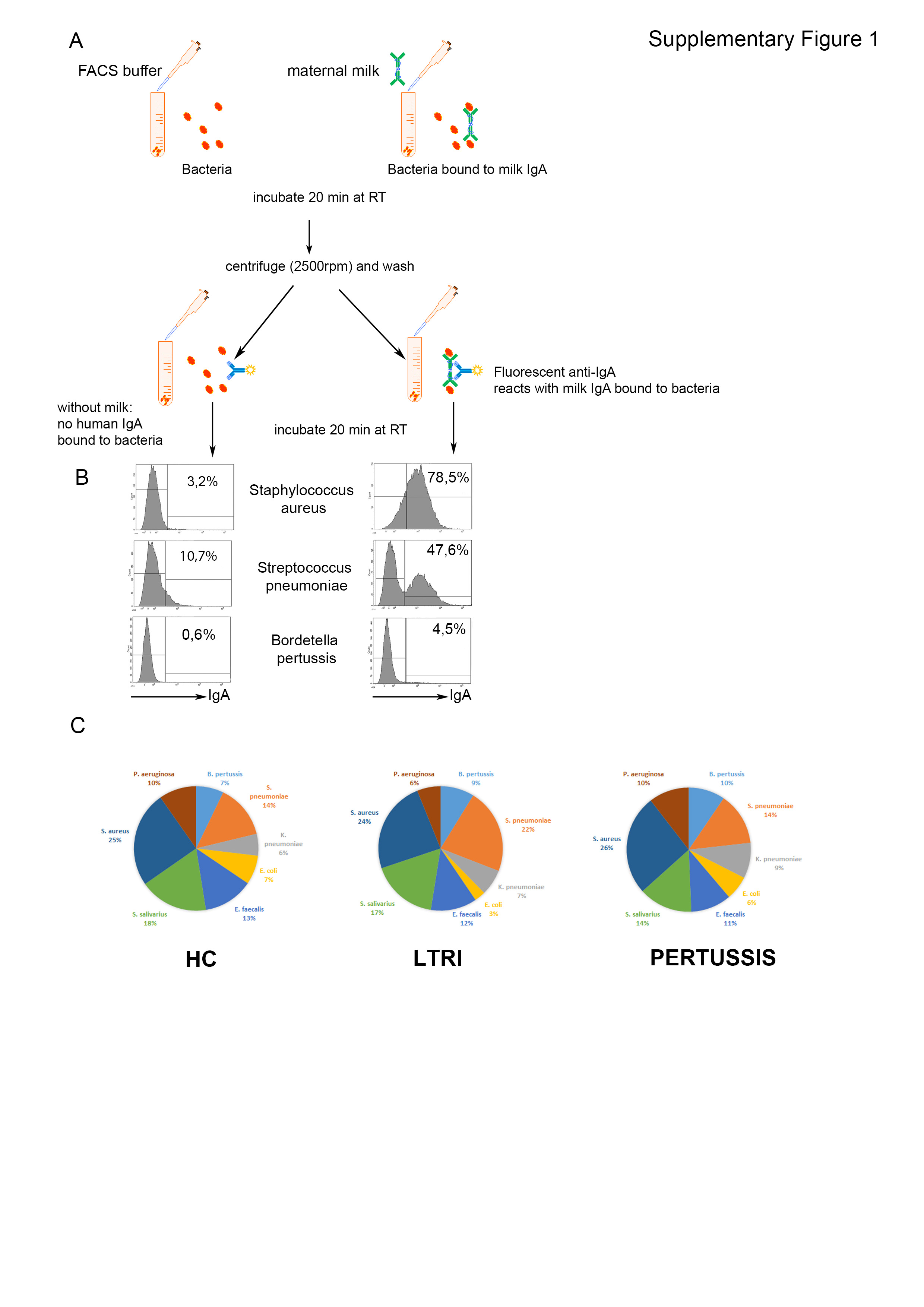

Supplement: Figure S1 — Description of the bacterial FACS method. (A) Colonies of bacterial isolates were diluted in FACS buffer and incubated either with FACS buffer or with maternal milk (1:10 and 1:30 dilutions). After washing, IgA bound to bacterial cells was revealed by a secondary staining with FITC-labeled mouse anti-human IgA. (B) Representative histograms showing the fluorescence of bacteria, without or with bound maternal IgA. (C) Relative representation of IgA binding to the indicated bacteria in the milk of healthy controls (HC), lower respiratory tract infections (LRTI), and PERTUSSIS mothers. As compared to B. pertussis, significantly higher amounts of antibodies bound to S. Aureus, S. salivarius, and S. pneumoniae in all of the three groups. [file image_1.jpeg]
